# Postangiography Prediction of Renal Replacement Therapy in Acute Myocardial Infarction–Related Cardiogenic Shock: Least Absolute Shrinkage and Selection Operator Nomogram Development and Validation

**DOI:** 10.2196/79678

**Published:** 2026-05-20

**Authors:** Amin Daoulah, Julia M Ladna, Abdulrahman Arabi, Ahmed Elmahrouk, Shaber Seraj, Hatem M Aloui, Mohammed Alshehri, Wael Almahmeed, Prashanth Panduranga, Nooraldaem Yousif, Amir Lotfi, Mohammed Qutub, Waleed Alharbi, Rajesh Rajan, Mokhtar Abdirahman Kahin, Abdullah Alenezi, Said Al Maashani, Taher Hassan, Jassim Alswuaidi, Fakhreldein Eltaieb, Mubarak Abdulhadi Aldossari, Mohammed Al Jarallah, Abdelmaksoud Elganady, Badr Alzahrani, Abdulrahman Alqahtani, Faisal Omar M Al Nasser, Husam A Noor, Mohamed Nabil Alama, Alaa Aldossari, Sultan Al Obaikan, Alsayed Ali Almarghany, Omar Kanbr, Youssef Elmahrouk, Ibrahim A M Abdulhabeeb, Mohammed Balghith, Ahmad S Hersi, Harvey Antony, Adnan Fathey Hussien, Abdulrahman Almoghairi, Mohamed Mohammednabil A Alama, Mohamed Ajaz Ghani, Hazim Rahbi, Ayman Basardah, Bandar Alshehri, Abdulhadi Alama, Laura AlObaid, Seraj Abualnaja, Tarique Shahzad Chachar, Hassan Khan, Shahrukh Hashmani, Ahmed A Ghonim, Khalid Almerri, Razan Alsofayan, Abdelaziz M Tawengi, Abeer M Shawky, Ali Alshehri, Amr Arafat, Ahmed Jamjoom

**Affiliations:** 1 King Faisal Specialist Hospital & Research Centre, Jeddah, Makkah, 23423, Saudi Arabia, 966 555042073; 2 University of Massachusetts Chan Medical School, Worcester, MA, United States; 3 Hamad Medical Corporation, Doha, Qatar; 4 Tanta University, Tanta, Gharbiya, Egypt; 5 King Saud Medical City, Riyadh, Saudi Arabia; 6 Armed Forces Hospital Southern Region, Khamis Mushait, Saudi Arabia; 7 Heart & Vascular Institute, Cleveland Clinic Abu Dhabi, Abu Dhabi, United Arab Emirates; 8 Department of Cardiology, National Heart Center, Royal Hospital, Muscat, Oman; 9 Department of Cardiology, Mohammed Bin Khalifa Specialist Cardiac Center, Awali, Bahrain; 10 King Abdulaziz University, Jeddah, Saudi Arabia; 11 King Saud University, Riyadh, Saudi Arabia; 12 Amiri Hospital, Sharq, Kuwait, Kuwait; 13 International Medical Center, Jeddah, Makkah, Saudi Arabia; 14 Chest Diseases Hospital, Shuwaikh, Kuwait; 15 Sultan Qaboos Hospital, Salalah, Oman; 16 Bugshan Hospital, Jeddah, Saudi Arabia; 17 Al-Azhar University, Cairo, Egypt; 18 Dr Erfan and Bagedo General Hospital, Jeddah, Saudi Arabia; 19 Prince Sultan Cardiac Center, Riyadh, Saudi Arabia; 20 King Fahad Medical City, Riyadh, Saudi Arabia; 21 Department of Cardiology, Madinah Cardiac Center, Madinah, Saudi Arabia; 22 King Abdulaziz Medical City, Riyadh, Saudi Arabia; 23 Elrazi University, Khartoum, Sudan; 24 Umm al-Qura University, Makkah, Saudi Arabia; 25 Departement of Internal Medicine, Al Salama Hospital, Jeddah, Saudi Arabia; 26 Department of Cardiology, King Abdulaziz Specialist Hospital, Sakaka, Saudi Arabia; 27 King Saud bin Abdulaziz University for Health Sciences, Riyadh, Saudi Arabia; 28 Royal College of Surgeons in Ireland, Dublin, Ireland; 29 King Khalid University, Abha, Saudi Arabia; 30 Research and Innovation Institute, Ministry of Defense Health Services, Riyadh, Saudi Arabia

**Keywords:** myocardial infarction, acute myocardial infarction, cardiogenic shock, renal replacement therapy, prediction model, Least Absolute Shrinkage and Selection Operator, LASSO, machine learning

## Abstract

**Background:**

Acute kidney injury critically impacts outcomes in cardiogenic shock secondary to acute myocardial infarction (CS-AMI). Acute kidney injury is one of the strongest independent predictors of in-hospital mortality in CS-AMI. Despite evidence that early renal replacement therapy (RRT) initiation improves survival, comprehensive prediction models for RRT in this population remain lacking.

**Objective:**

This study aimed to develop and internally validate a Least Absolute Shrinkage and Selection Operator (LASSO) regression–based prediction model and clinical nomogram for in-hospital RRT in patients with CS-AMI.

**Methods:**

This multicenter retrospective cohort study included 1431 patients with CS-AMI from the Gulf Cardiogenic Shock (Gulf-CS) registry across 13 centers in 6 Gulf countries (2020‐2022). LASSO logistic regression was applied to a training set (1071/1431, 80%) to select baseline predictors of RRT; performance was evaluated on a held-out testing set (268/1431, 20%). Internal validation included 10-fold cross-validation and bootstrapping (1000 iterations). Cluster-robust SEs accounted for center effects. The model was compared to a parsimonious model (age+creatinine clearance), and a clinical nomogram was developed.

**Results:**

Of 1431 patients, 190 (13.3%) required RRT. Patients requiring RRT were significantly older (mean 64.17, SD 12.14 y vs mean 59.75, SD 11.77 y; *P*<.001), with higher prevalences of diabetes mellitus (72.1% vs 61.9%; *P*=.008), peripheral arterial disease (11.6% vs 3.7%; *P*<.001), and prior cerebrovascular accident (11.1% vs 5.7%; *P*=.005). The RRT group had lower creatinine clearance (46 vs 72 mL/min; *P*<.001), higher baseline lactate (2.7 vs 2.1 mmol/L; *P*<.001), and more advanced Society for Cardiovascular Angiography and Interventions (SCAI) shock stages (stages D and E: 90.5% vs 64.9%; *P*<.001). LASSO selected 15 baseline predictors. The model achieved an area under the receiver operating characteristic curve (AUC) of 0.714 on the testing set, significantly outperforming the parsimonious model (AUC: 0.624; *P*<.001). Bootstrap-corrected AUC was 0.745 (95% CI 0.730‐0.756). In-hospital mortality was markedly higher in the RRT group (75.8% vs 38.8%; *P*<.001), with longer hospital stay (10 vs 6 d; *P*<.001), more major bleeding (16.8% vs 7.3%; *P*<.001), and cerebrovascular accidents (11.1% vs 4.9%; *P*=.001).

**Conclusions:**

We have developed and internally validated a robust 15-variable nomogram (Gulf-CS-Nomogram) that accurately predicts the need for RRT in patients with CS-AMI using baseline data intended for use after coronary angiography. This tool may facilitate early nephrology consultation and timely RRT initiation to improve outcomes.

## Introduction

Cardiogenic shock (CS) is an end-organ damage and tissue hypoxia caused by cardiac dysfunction, with acute myocardial infarction (AMI) being the most common underlying cause. AMI leads to myocardial necrosis, loss of ventricular function, and reduced cardiac output, making CS a leading cause of in-hospital mortality in patients with AMI [1]. Within the United States, the incidence of CS in patients with AMI ranges from 5% to 10%, and despite advances in treatment, the incidence has been increasing since 2000 [2,3]. Mortality remains high, with 30-day mortality rates of approximately 40% and 1-year mortality rates approaching 50%. Survivors of CS often experience significant morbidity, including high rates of readmission and the need for ongoing supportive care [1].

Acute kidney injury (AKI) is a common and severe complication in patients with cardiogenic shock secondary to acute myocardial infarction (CS-AMI). In this context, the primary driver of AKI is reduced cardiac output, leading to decreased renal perfusion and increased renal venous pressure [4,5]. Additional contributors include microvascular injury and hemodynamic instability [6]. Studies report AKI incidence rates ranging from 35% to 55% in patients with CS-AMI, with approximately 13% requiring renal replacement therapy (RRT) [6,7]. AKI is associated with worse outcomes, including increased in-hospital mortality, longer lengths of stay, and higher health care costs. Notably, AKI is the strongest independent predictor of in-hospital mortality in patients with CS-AMI [8,9]. Lauridsen et al [7] reported that AKI requiring RRT in patients with CS-AMI was associated with a 62% in-hospital mortality rate, and survivors faced a significantly greater risk of chronic dialysis than patients with non-AKI (11% vs 1%).

Several studies have highlighted the potential benefits of early initiation of RRT in critically ill patients. For example, the Early versus Late Initiation of RRT in Critically Ill Patients with Acute Kidney Injury (ELAIN) trial demonstrated that early RRT initiation significantly reduced 90-day mortality compared with delayed RRT initiation (39.3% vs 54.7%) and improved renal recovery [10]. Similarly, a meta-analysis by Zou et al [11] revealed that early RRT reduced 30-day mortality and shortened the length of stay in postcardiac surgery patients with AKI.

These findings highlight the critical impact of AKI on prognosis and early intervention on mortality in patients with CS-AMI. Few studies have identified risk factors or predictors for RRT in this population. Some known predictors include a reduced glomerular filtration rate at admission, elevated central venous pressure, decreased mixed venous oxygen saturation, increased age, increased comorbidity burden, and preexisting chronic kidney disease [9,12]. Comprehensive models predicting the need for RRT in patients with CS-AMI are still needed. In this study, we aimed to predict RRT in this population via Least Absolute Shrinkage and Selection Operator (LASSO) logistic regression. Predicting the need for RRT could help reduce mortality and improve outcomes.

## Methods

### Study Population

The Gulf Cardiogenic Shock (Gulf-CS) registry is a multicenter retrospective registry of CS secondary to angiographically confirmed myocardial infarction [13]. The registry included 1513 patients from 13 tertiary referral centers in 6 Gulf countries (Saudi Arabia, Qatar, Oman, the United Arab Emirates, Kuwait, and Bahrain) between January 2020 and December 2022. Data on baseline demographics, comorbidities, number of pressors or inotropes, angiographic findings, hemodynamic measurements, and mechanical circulatory devices were collected.

Patients with baseline creatinine clearance <15 mL/minute and those with chronic kidney disease requiring dialysis at presentation were excluded. For this analysis, we divided the patients into 2 groups based on their need for RRT: those who did not require RRT (n=1241) and those who required RRT (n=190). We compared the two groups’ baseline variables, angiographic findings, treatment, and in-hospital outcomes. RRT used in this study was hemodialysis (n=54), peritoneal dialysis (n=1), and continuous venovenous hemodialysis (n=135).

### Study Outcomes

Hospital outcomes were compared between patients with and without RRT. The primary outcome was in-hospital all-cause mortality. The secondary outcomes included revascularization, cerebrovascular accident (CVA), the occurrence of major or minor bleeding, and the duration of hospital stay.

### Study Definitions

CS was defined according to the Standardized Definitions for Cardiogenic Shock Research and Mechanical Circulatory Support Devices as follows: (1) systolic blood pressure <90 mm Hg for ≥30 minutes or the need for vasopressors, inotropes, or mechanical circulatory support (MCS) to maintain a systolic blood pressure ≥90 mm Hg and (2) evidence of tissue hypoperfusion [14]. The time of shock onset is the time when CS is first diagnosed. CVAs include stroke (ischemic or hemorrhagic) and transient ischemic attack. Bleeding events were defined according to the Bleeding Academic Research Consortium (BARC) classification [15]. Creatinine clearance was calculated using the Cockcroft-Gault equation: CrCl=[(140 – age)×weight (kg) × (0.85 if female)]/[72×serum creatinine (mg/dL)]. We chose this method over estimated glomerular filtration rate equations because of its established use in the CS literature, its incorporation of body weight (relevant to acute volume shifts), and its direct applicability to medication dosing decisions. Right ventricular (RV) dysfunction was defined by echocardiographic criteria: tricuspid annular plane systolic excursion <17 mm, S’ wave <12 cm/s, and tricuspid annular plane systolic excursion to pulmonary artery systolic pressure ratio <0.34 [16]. Baseline data, including laboratory and echocardiographic data, were measured at the time of hospital presentation. The number of diseased vessels was defined as the total count of coronary arterial branches with angiographically significant stenosis (≥50% luminal obstruction), including major epicardial vessels and their principal branches (left anterior descending artery, diagonal branches, obtuse marginal branches, ramus intermedius, right coronary artery, posterior descending artery, and posterolateral branches), yielding a possible range of 1 to 6 in this cohort. Details of the Gulf-CS registry and variable definitions have already been published [13].

### Ethical Considerations

The Institutional Review Board (IRB) of King Faisal Specialist Hospital and Research Center in Jeddah approved the study on January 16, 2023 (IRB 2022‐90: Gulf-CS Registry). Approval was obtained from each center’s ethics committee for all study sites. The IRB waived the need for patient consent due to the study’s observational nature. Patients' privacy was maintained throughout the study, and deidentified data were shared. No compensation was provided to participants.

### Statistical Analysis

Statistical analysis was conducted with Stata 18 (StataCorp) and Python 3.11 (Python Software Foundation). Continuous variables were summarized using means (SDs) or medians (Q1-Q3) according to the normal distribution. Categorical data are reported as numbers (%). Comparisons between the two groups were made using *t* tests, Mann-Whitney tests, chi-square tests, or Fisher exact tests, as appropriate. Missing data were handled using complete case analysis.

### Model Development and Variable Selection

The dataset was randomly partitioned into a training set (80%) and a testing set (20%), stratified by the primary outcome of in-hospital RRT. We used the LASSO logistic regression on the training set to identify the most parsimonious set of baseline predictors. The LASSO method applies a penalty that shrinks the coefficients of less important variables toward zero, effectively performing automated variable selection. The tuning parameter (lambda) was selected via 10-fold cross-validation to maximize the area under the receiver operating characteristic curve (AUC).

### Model Performance and Validation

The model’s discrimination was assessed on the held-out test set using the AUC. We performed two forms of internal validation:

1. 10-fold cross-validation on the training set.
2. Bootstrapping with 1000 resamples on the training set to generate a bias-corrected 95% CI for the AUC.

Model calibration was assessed visually by plotting the observed versus predicted probabilities on the testing set.

### Model Calibration

Model calibration was assessed visually by plotting the observed versus predicted probabilities on the testing set. To improve calibration, we applied Platt scaling (also known as sigmoid calibration) to the LASSO model predictions. Platt scaling fits a logistic regression model to the predicted probabilities from the LASSO model, effectively recalibrating the predictions to better match the observed event rates. This post hoc calibration was performed using 5-fold cross-validation on the training set, and the calibrated model was then applied to the testing set. Calibration performance was evaluated both before and after Platt scaling using calibration plots.

### Nomogram Development

A clinical nomogram was constructed to provide individualized, point-based estimates of the probability of requiring RRT. The nomogram was derived from the LASSO logistic regression model described above, using the penalized coefficients of all 15 candidate variables retained at the optimal regularization parameter. To facilitate clinical application, the standardized LASSO coefficients were backtransformed to their original measurement scales by dividing each coefficient by the corresponding variable’s SD, with continuous variables expressed in their natural clinical units. For each predictor, a point score proportional to its backtransformed coefficient and clinical range was assigned relative to the variable with the largest absolute contribution; the sum of individual point scores maps to a predicted probability of RRT via the logistic function. The nomogram is intended as a bedside decision-support tool to stratify patients with CS-AMI by their risk of acute kidney injury requiring RRT using variables available at or shortly after admission.

### Addressing Center Effect and Model Comparison

To account for potential clustering of outcomes by institution, we fitted a final logistic regression model using the LASSO-selected variables with cluster-robust SEs for each center. Continuous variables were entered into the final model as unstandardized values to yield clinically interpretable odds ratios (ORs). The Society for Cardiovascular Angiography and Interventions (SCAI) shock stage was treated as a categorical variable with stage B as the reference category, as this approach more accurately captures the nonlinear risk transitions between stages. To demonstrate the clinical utility of our model, we compared its performance on the test set against a parsimonious model that included only age and baseline creatinine clearance.

### Multicollinearity

We assessed multicollinearity among the predictors in the final model using the variance inflation factor (VIF). A VIF >5 was considered indicative of significant multicollinearity. All included features had VIF <1.5.

## Results

### Baseline Characteristics

The baseline characteristics of the patients in our study revealed several demographic and clinical differences between those who required RRT and those who did not. The cohort’s mean age was 60.34 (SD 12.14) years, with patients requiring RRT significantly older (mean 64.17, SD 12.14 y) than those who did not (mean 59.75, SD 11.77 y; *P*<.001). Males comprised most of the cohort (1162/1431, 81.20%), with no significant difference between the 2 groups (*P*=.46). Patients who required RRT had a greater prevalence of comorbid conditions, including diabetes mellitus (72.11% vs 61.89%; *P*=.008), peripheral arterial disease (PAD; 11.58% vs 3.71%; *P*<.001), a history of CVA (11.05% vs 5.72%; *P*=.005), and congestive heart failure (15.79% vs 10.64%; *P*=.04). Other comorbidities, such as dyslipidemia, hypertension, previous myocardial infarction, and history of coronary interventions (percutaneous coronary intervention [PCI] and coronary artery bypass grafting [CABG]), were similar between the groups. Patients requiring RRT presented with more severe disease, as evidenced by higher rates of anterior ST-elevation myocardial infarction (STEMI) and cardiac arrest at presentation. Additionally, RV dysfunction was more prevalent among patients requiring renal support (23.28% vs 15.76%; *P*=.01), further highlighting the increased severity of their clinical presentation.

Laboratory investigations revealed lower hemoglobin, creatinine clearance, sodium bicarbonate levels, and higher potassium levels in patients requiring RRT. Patients who required RRT had a higher SYNTAX score, a greater number of vessels with significant coronary disease, and an advanced SCAI stage than patients who did not require RRT (Table 1).

**Table 1.** Comparison of the baseline data between patients who did and did not need renal replacement therapy.

Variables	Total (N=1431)	No renal replacement therapy (n=1241)	Renal replacement therapy (n=190)	*P* value
Age (years), mean (SD)	60.34 (12.14)	59.75 (11.77)	64.17 (12.14)	<.001
Males, n (%)	1162 (81.20)	1004 (80.90)	158 (83.16)	.46
BMI (kg/m^2^), median (IQR)	27 (24‐30)	27 (24‐30)	27 (25‐29)	.58
Diabetes mellitus, n (%)	905 (63.24)	768 (61.89)	137 (72.11)	.008
Dyslipidemia, n (%)	811 (56.67)	701 (56.49)	110 (57.89)	.72
Hypertension, n (%)	891 (62.26)	761 (61.32)	130 (68.42)	.60
Previous myocardial infarction, n (%)	320 (22.36)	275 (22.16)	45 (23.68)	.64
History of PCI^a^, n (%)	283 (18.78)	245 (19.74)	38 (20)	.93
History of CABG^b^, n (%)	47 (3.28)	39 (3.14)	8 (4.21)	.44
Peripheral arterial disease, n (%)	68 (4.75)	46 (3.71)	22 (11.58)	<.001
History of CVA^c^, n (%)	92 (6.43)	71 (5.72)	21 (11.05)	.005
History of CHF^d^, n (%)	162 (11.32)	132 (10.64)	30 (15.79)	.04
COPD^e^, n (%)	47 (3.28)	43 (3.46)	4 (2.11)	.33
Presentation, n (%)				
STEMI^f^	1061 (74.14)	948 (76.39)	113 (59.47)	<.001
NSTEMI^g^	370 (25.86)	293 (23.61)	77 (40.53)	<.001
Cardiac arrest	789 (55.14)	657 (52.94)	132 (69.47)	<.001
Tachyarrhythmia	450 (31.49)	381 (30.75)	69 (36.32)	.12
Bradyarrhythmia	202 (14.12)	169 (13.62)	33 (17.37)	.16
ECG^h^, n (%)				
Location of STEMI				<.001
NSTEMI	370 (25.86)	293 (23.61)	77 (40.53)	
Anterior	689 (48.15)	611 (49.23)	78 (41.05)	
Lateral	321 (22.43)	290 (23.37)	31 (16.32)	
Inferior	51 (3.56)	47 (3.79)	4 (2.11)	
Echocardiography				
Ejection fraction, median (IQR)	32 (23‐39)	32 (23‐39)	33 (24‐38)	.13
Right ventricular dysfunction, n (%)	237 (16.76)	193 (15.76)	44 (23.28)	.01
PASP^i^ (mm Hg), median (IQR)	35 (30‐40)	35 (30‐40)	35 (31‐40)	.16
Laboratory data, median (IQR)				
Hemoglobin (g/dL)	13.3 (11.7‐14.9)	13.5 (12-15)	12.3 (10.9‐14)	<.001
WBCs^j^ (10^9^L)	12.4 (9.8‐15.8)	12.4 (9.8‐15.3)	13 (10‐16.4)	.20
Potassium (mmol/L)	4.2 (3.8‐4.6)	4.2 (3.8‐4.6)	4.4 (3.98‐4.8)	.001
Creatinine clearance (mL/min)	68 (43‐95)	72 (47‐97)	46 (33‐74)	<.001
Baseline lactate (mmol/L)	2.2 (1.3‐3.8)	2.1 (1.2‐3.5)	2.7 (1.9‐4.6)	<.001
HCO_3_ (mEq/L)	20 (17‐23)	20 (17‐23)	18 (17‐22)	<.001
Angiography, median (IQR)				
SYNTAX score	25 (19‐32)	25 (19‐32)	28 (22‐33)	<.001
Vessels with significant disease	3 (2-3)	1 (2-3)	3 (2-4)	<.001
SCAI^k^ stage, n (%)				<.001
B	91 (6.36)	90 (7.25)	1 (0.53)	
C	363 (25.37)	346 (27.88)	17 (8.95)	
D	704 (49.20)	596 (48.03)	108 (56.84)	
E	273 (19.08)	209 (16.84)	64 (33.68)	

a PCI: percutaneous coronary intervention.

b CABG: coronary artery bypass grafting.

c CVA: cerebrovascular accident.

d CHF: congestive heart failure.

e COPD: chronic obstructive pulmonary disease.

f STEMI: ST-elevation myocardial infarction.

g NSTEMI: non–ST-elevation myocardial infarction.

h ECG: electrocardiogram.

i PASP: pulmonary artery systolic pressure.

j WBC: white blood cell.

k SCAI: Society for Cardiovascular Angiography and Interventions.

### CS Management

Inotropic support, MCS, and mechanical ventilation were used more frequently in patients who needed renal support. CABG was performed more frequently in patients requiring RRT, and PCI was more common in patients without RRT (Table 2).

**Table 2.** Comparison of the management data between patients who did and did not need renal replacement therapy.

Variables	Total (N=1431)	No renal replacement therapy (n=1241)	Renal replacement therapy (n=190)	*P* value
Pressor or inotropic support, n (%)	1294 (90.43)	1110 (89.44)	184 (96.84)	.001
Mechanical circulatory support, n (%)	660 (46.12)	529 (42.63)	131 (68.95)	<.001
Intraaortic balloon	503 (33.25)	399 (30.95)	104 (46.43)	
ECMO^a^	27 (1.78)	23 (1.78)	4 (1.79)	
Impella	31 (2.05)	26 (2.02)	5 (2.23)	
IABP^b^+ ECMO	95 (6.28)	71 (5.51)	24 (10.71)	
IABP+ Impella	12 (0.79)	8 (0.62)	4 (1.79)	
ECMO+ Impella	10 (0.66)	6 (0.47)	4 (1.79)	
IABP+ ECMO+Impella	15 (0.99)	11 (0.85)	4 (1.79)	
Mechanical ventilation, n (%)	878 (61.36)	702 (56.57)	176 (92.63)	<.001
Treatment, n (%)				.007
Medical	137 (9.57)	118 (9.51)	19 (10)	
PCI^c^	1174 (82.04)	1030 (83)	144 (75.79)	
CABG^d^	120 (8.39)	93 (7.49)	27 (14.21)	

a ECMO: extracorporeal membrane oxygenation.

b IABP: intraaortic balloon pump.

c PCI: percutaneous coronary intervention.

d CABG: coronary artery bypass grafting.

### Hospital Outcomes

Hospital mortality (76% vs 39%; *P*<.001) and CVA (11% vs 5%, *P*=.001) were significantly greater in patients with RRT. Hospitalization duration was prolonged in patients with RRT (10 vs 6 d; *P*<.001). No differences were found in revascularizations or minor bleeding between the groups (Table 3).

**Table 3.** Comparison of hospital outcomes between patients who did and did not need renal replacement therapy.

Hospital outcomes	Total (N=1431)	No renal replacement therapy (n=1241)	Renal replacement therapy (n=190)	*P* value
Hospital mortality, n (%)	625 (43.68)	481 (38.76)	144 (75.79)	<.001
Target lesion revascularization, n (%)	57 (3.98)	49 (3.95)	8 (4.21)	.86
Target vessel revascularization, n (%)	43 (3)	37 (2.98)	6 (3.16)	.89
Cerebrovascular accident, n (%)	82 (5.73)	61 (4.92)	21 (11.05)	.001
Major bleeding, n (%)	123 (8.60)	91 (7.33)	32 (16.84)	<.001
Minor bleeding, n (%)	27 (1.89)	23 (1.85)	4 (2.11)	.77
Duration of hospital stay (d), median (IQR)	6 (3-13)	6 (2-12)	10 (4‐22)	<.001

### LASSO Model Development

**Figure 1.**
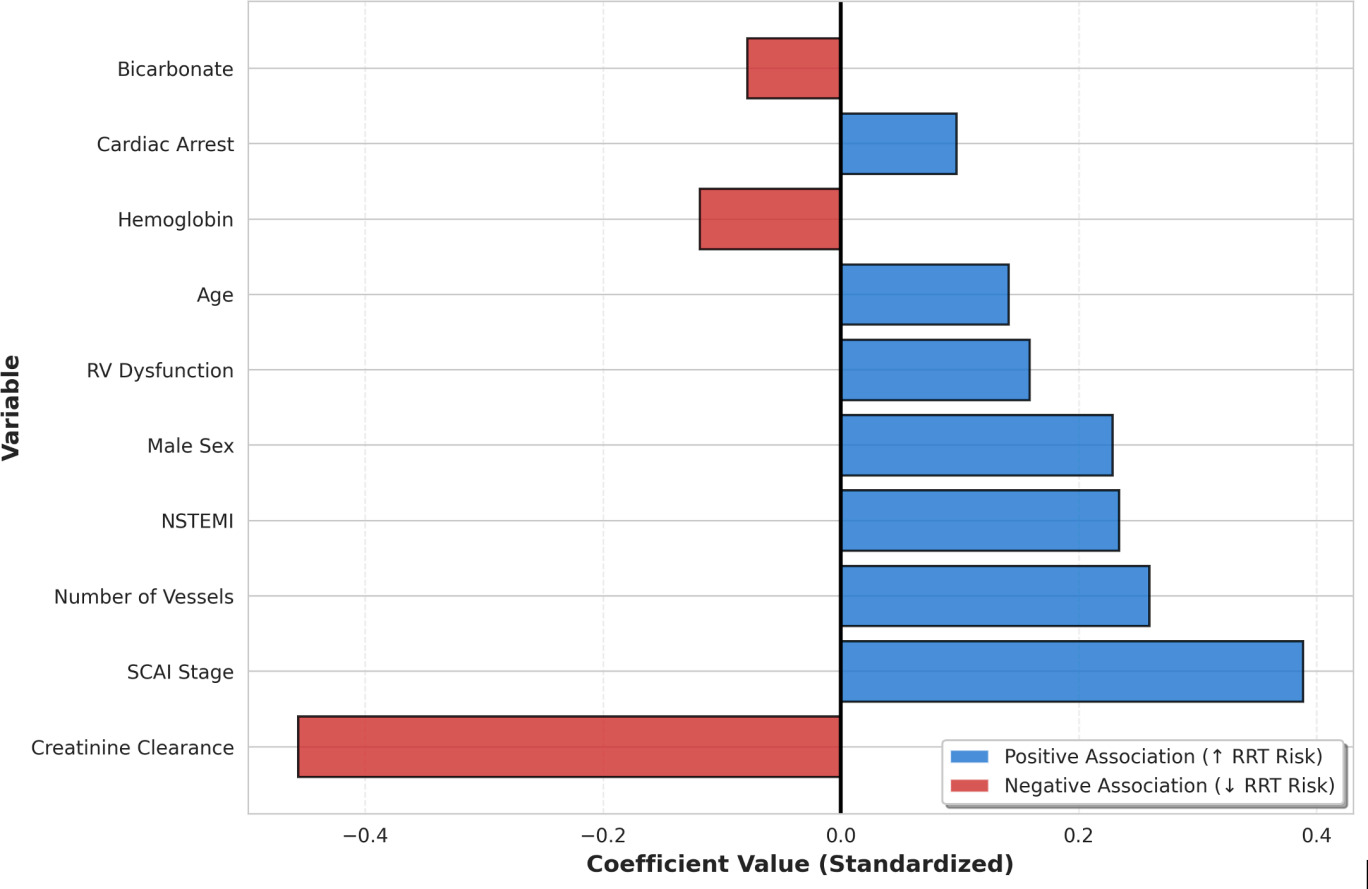
Least Absolute Shrinkage and Selection Operator–selected variable coefficients for renal replacement therapy prediction. Top 10 baseline predictors ranked by absolute coefficient value. Blue bars: positive association with renal replacement therapy risk; red bars: negative association with renal replacement therapy risk. All coefficients are standardized. NSTEMI: non–ST-elevation myocardial infarction; RRT: renal replacement therapy; RV: right ventricular; SCAI: Society for Cardiovascular Angiography and Interventions.

The LASSO model included 1072 in the training dataset and 268 in the test dataset (a total of 1340), while 91 patients were dropped due to missing baseline data. From the baseline variables, the LASSO regression model selected a final set of 15 predictors for RRT. The selected variables and their corresponding coefficients are shown in Figure 1. The 3 strongest predictors were lower creatinine clearance, higher SCAI shock stage, and a greater number of diseased coronary vessels (Table S1 in Multimedia Appendix 1).

### Model Performance

**Figure 2.**
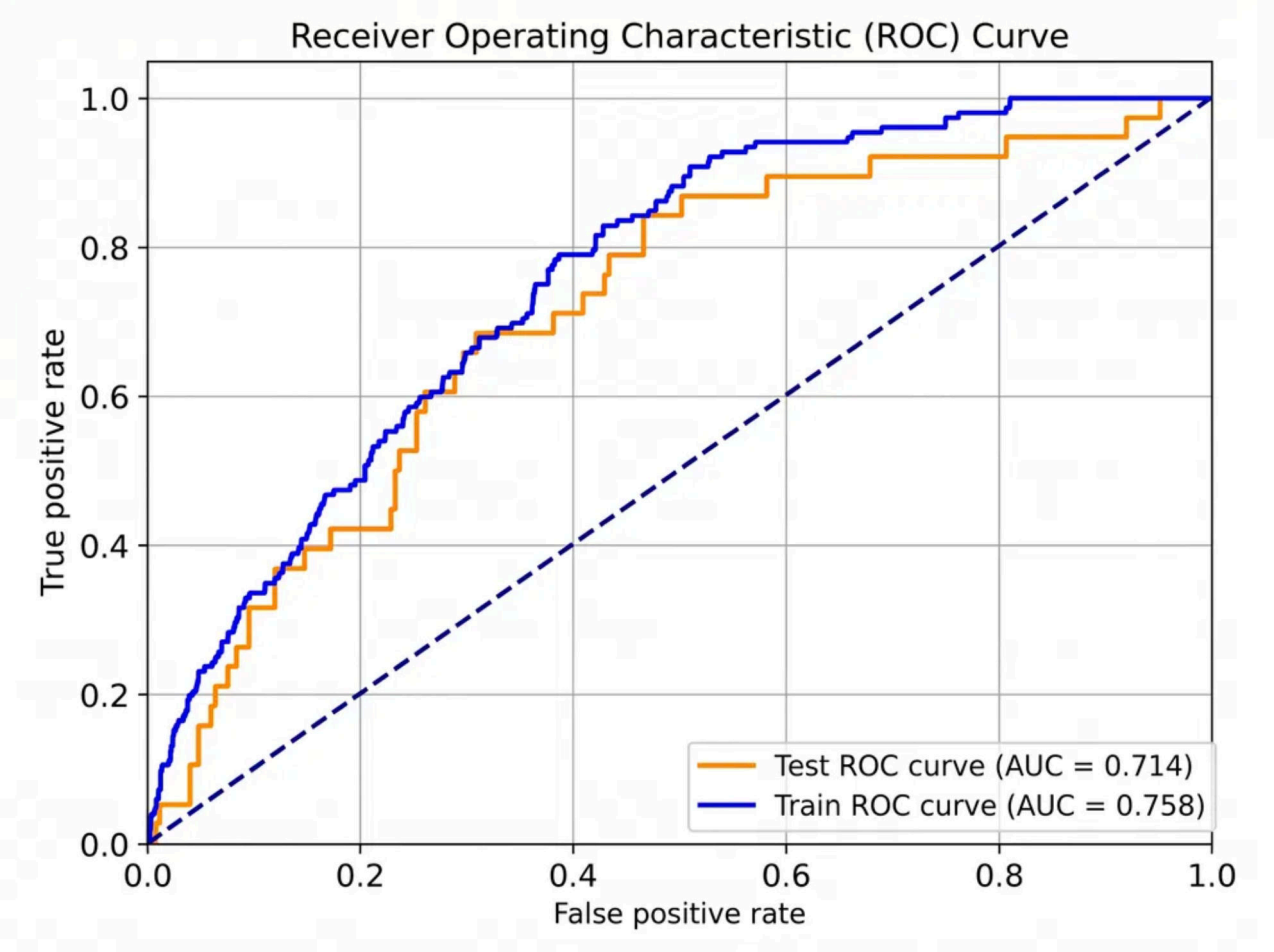
Receiver operating characteristic curves for the Least Absolute Shrinkage and Selection Operator prediction model. Blue curve: training set (AUC=0.758, n=1072); orange curve: testing set (AUC=0.714, n=268). Dashed line: reference (AUC=0.50). The model demonstrated good discrimination with minimal overfitting. AUC: area under the receiver operating characteristic curve; ROC: receiver operating characteristic.

**Figure 3.**
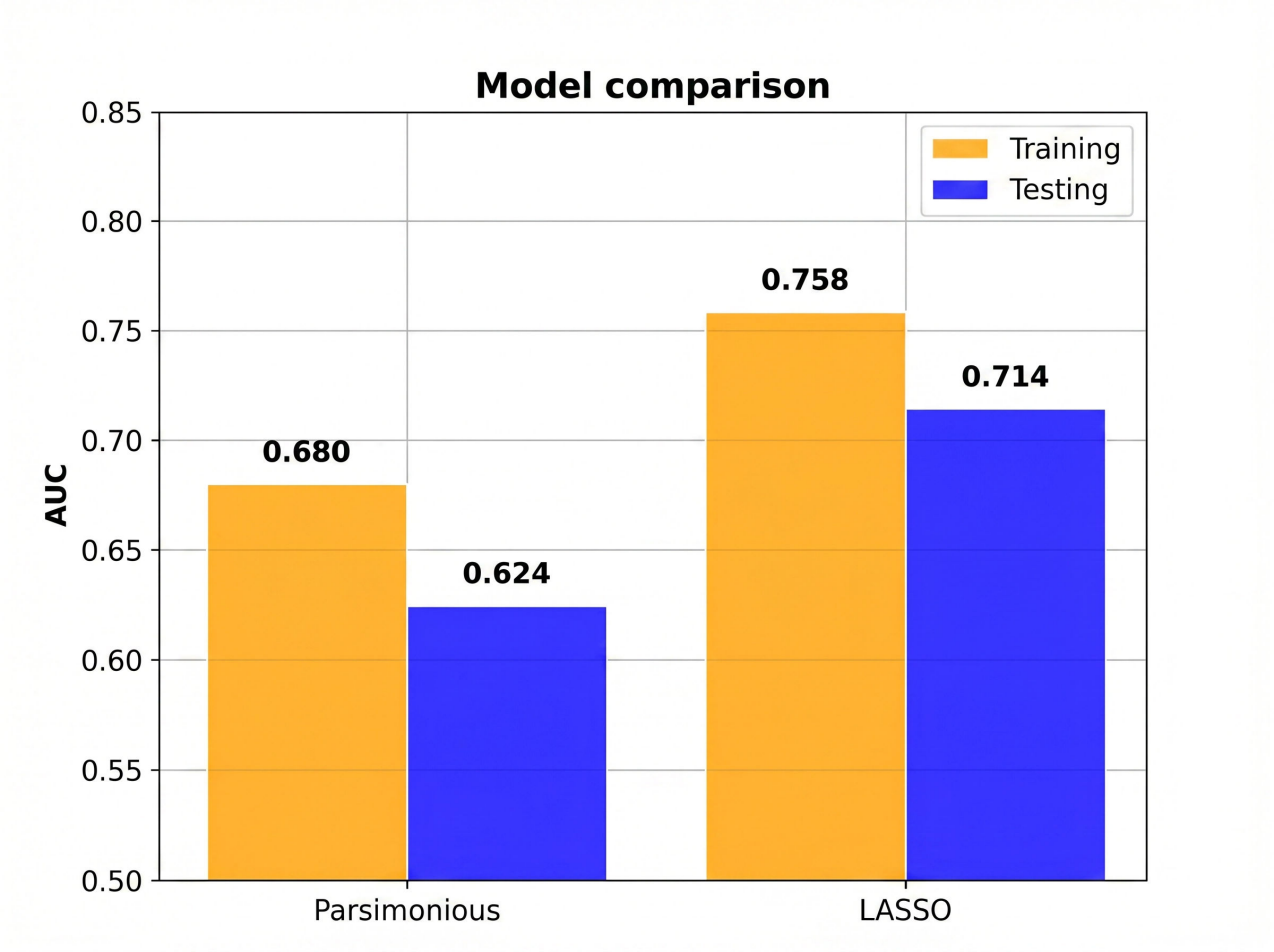
Model performance comparison. Orange bars: training set (n=1072); blue bars: testing set (n=268). The 15-variable Least Absolute Shrinkage and Selection Operator model (AUC=0.714) significantly outperformed the 2-variable parsimonious model containing only age and creatinine clearance (AUC=0.624), with an improvement of +0.090 AUC points (*P*<.001). AUC: area under the receiver operating characteristic curve; LASSO: Least Absolute Shrinkage and Selection Operator.

The final model demonstrated good discrimination on the held-out testing set, with an AUC of 0.714 (Figure 2). This was significantly better than the parsimonious model containing only age and creatinine clearance, which had an AUC of 0.624 (*P*<.001 for the difference; Figure 3). The model was well-calibrated, as shown in the calibration plot (Figure S1 in Multimedia Appendix 2).

### Internal Validation

The model’s performance was confirmed through 2 internal validation methods. A 10-fold cross-validation on the training set yielded a mean AUC of 0.723 (SD 0.050). Furthermore, bootstrapping with 1000 iterations produced a bias-corrected AUC of 0.745 (95% CI 0.730‐0.756), indicating robust and stable performance (Figure S2 in Multimedia Appendix 3).

### Nomogram

**Figure 4.**
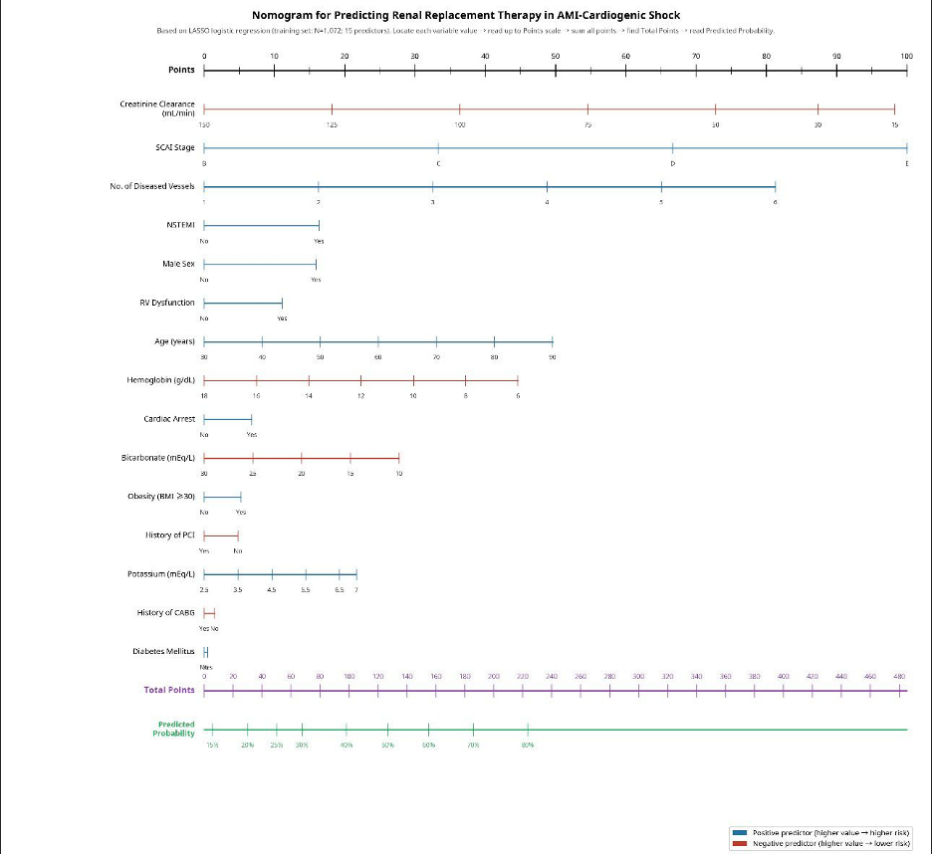
Gulf Cardiogenic Shock Nomogram for predicting in-hospital renal replacement therapy in patients with acute myocardial infarction complicated by cardiogenic shock. The nomogram was derived from the Least Absolute Shrinkage and Selection Operator logistic regression model. For each variable, the patient’s value is located on the corresponding row and projected vertically to the points scale (0‐100). Individual points are summed to obtain total points, which are then projected to the predicted probability of renal replacement therapy scale. Blue axes indicate positive associations with renal replacement therapy risk; red axes (creatinine clearance, hemoglobin, bicarbonate, history of percutaneous coronary intervention, and history of coronary artery bypass grafting) indicate negative associations, with axes running right to left such that lower values (higher risk) correspond to more points. CABG: coronary artery bypass grafting; NSTEMI: non–ST-segment elevation myocardial infarction; PCI: percutaneous coronary intervention; RV: right ventricular; SCAI: Society for Cardiovascular Angiography and Interventions.

A nomogram was developed based on the final LASSO model (Figure 4). A nomogram can be used post angiography immediately to estimate an individual patient’s probability of requiring in-hospital RRT by summing the points assigned to each predictor variable and reading the corresponding probability from the total points scale.

### Multivariable Analysis With Cluster-Robust SEs

In the final logistic regression model accounting for center-level clustering, several predictors remained significantly associated with the need for RRT. These included history of PCI (OR 0.649; *P*=.007), non–ST-elevation myocardial infarction (NSTEMI) presentation (OR 1.756; *P*=.02), SCAI stage D (OR 10.859; *P*=.02) and E (OR 14.613; *P*=.005), lower hemoglobin (OR 0.892; *P*=.03), and increased number of diseased vessels (OR 1.351; *P*=.001; Table 4). No significant multicollinearity was detected among the predictors (all VIF <1.5).

**Table 4.** Multivariable logistic regression with cluster-robust SEs for prediction of in-hospital renal replacement therapy. Logistic regression model fitted across multiple centers (n=1340). Standard errors are adjusted for clustering by center. Society for Cardiovascular Angiography and Interventions stage B is the reference category.

Variable	Odds ratio (95% CI)	*P* value
Age (per 10-y increase)	1.178 (0.996‐1.393)	.06
Sex (male)	1.925 (0.923‐4.012)	.08
Obesity (BMI ≥30)	1.209 (0.922‐1.586)	.17
Diabetes mellitus	1.031 (0.773‐1.375)	.84
History of PCI^a^	0.649 (0.473‐0.891)	.007
History of CABG^b^	0.838 (0.343‐2.045)	.70
NSTEMI^c^ (vs STEMI^d^)	1.756 (1.081‐2.851)	.02
Cardiac arrest	1.613 (0.938‐2.772)	.08
RV^e^ dysfunction	1.506 (0.852‐2.665)	.16
SCAI^f^ stage C (vs B)	4.740 (0.596‐37.675)	.14
SCAI stage D (vs B)	10.859 (1.439‐81.924)	.02
SCAI stage E (vs B)	14.613 (2.232‐95.676)	.005
Hemoglobin (per 1 g/dL)	0.892 (0.804‐0.989)	.03
Potassium (per 1 mEq/L)	1.186 (0.934‐1.506)	.16
Bicarbonate (per 1 mEq/L)	0.975 (0.927‐1.025)	.32
Creatinine clearance (per 1 mL/min)	0.996 (0.986‐1.006)	.39
Number of diseased vessels	1.351 (1.181‐1.545)	.001

a PCI: percutaneous coronary intervention.

b CABG: coronary artery bypass grafting.

c NSTEMI: non–ST-segment elevation myocardial infarction.

d STEMI: ST-segment elevation myocardial infarction.

e RV: right ventricular.

f SCAI: Society for Cardiovascular Angiography and Interventions.

## Discussion

### Principal Findings

This study used data from the Gulf-CS registry, the first large multinational study focused on patients with CS-AMI from the Gulf region. This study explored hospital and short-term outcomes of patients with CS-AMI across 6 Gulf countries, using the novel CS staging system developed by the SCAI Cardiogenic Shock Working Group (SCAI-CSWG). We used LASSO regression to predict the need for RRT in this high-risk population. Our analysis identified several predictors of RRT, including reduced creatinine clearance, advanced SCAI stage, and significant coronary artery disease affecting multiple vessels.

Renal support was required in 13.28% of patients (190/1431), and those requiring RRT had a significantly worse prognosis. This finding is consistent with previous studies showing that the RRT incidence in patients with CS-AMI is approximately 13% [7,17]. Patients who required renal support were notably older, with a mean age of 64.17 (SD 12.14) years, whereas those who did not require RRT were 59.75 (SD 11.77) years (*P*<.001), which is also consistent with the literature [9,18]. Comorbidities were also more prevalent in the renal support group, with higher rates of diabetes mellitus (72.11% vs 61.89%; *P*=.008), PAD (11.58% vs 3.71%; *P*<.001), previous CVA (11.05% vs 5.72%; *P*=.005), and congestive heart failure (15.79% vs 10.64%; *P*=.037). The literature also highlights a high comorbidity burden associated with increased renal support needs [19]. Patients presenting with NSTEMI and those experiencing cardiac arrest were also more likely to require RRT. Specifically, 40.53% (77/190) of patients requiring RRT had NSTEMI, whereas 23.61% (293/1241) of patients in the no-renal-support group did (*P*<.001). Cardiac arrest was present in 69.47% (132/190) of patients in the RRT group, which was significantly greater than the 52.94% reported in patients who did not require RRT (*P*<.001). These findings highlight the association between more severe initial presentations and the subsequent need for RRT. In a study by Jeppesen et al [18], 33.5% of patients who experienced out-of-hospital cardiac arrest developed AKI, and 10.1% required RRT.

Regarding echocardiographic findings, RV dysfunction was significantly more prevalent in the renal support group, suggesting that RV failure and venous congestion contribute to worsening renal function. This finding is corroborated by Jain and associates, who demonstrated that RV dysfunction is common in patients with CS and is associated with increased in-hospital mortality [20]. RV failure is associated with poor outcomes and often requires MCS [21]. Furthermore, baseline laboratory marker levels, such as creatinine clearance and hemoglobin, potassium, and lactate levels, were significantly lower in patients who required RRT. The median creatinine clearance was markedly lower in the RRT group (46 mL/min vs 72 mL/min; *P*<.001), and the baseline lactate level was greater (2.7 mmol/L vs 2.1 mmol/L; *P*<.001), indicating more pronounced tissue hypoperfusion. This finding is consistent with other studies that associated worse laboratory abnormalities with renal support [22].

Finally, angiographic findings such as the SYNTAX score, number of major vessels involved, and SCAI stage were also obtained. The data suggested that the SYNTAX score was greater in the renal support group, with more significant coronary disease in the RRT group. This finding is consistent with prior findings that lesion complexity increases with declining renal function [23]. Significant differences in SCAI stages, with more severe stages (D and E), were also observed in the RRT group (*P*<.001).

### Mortality and Outcomes

The need for RRT was associated with a significantly higher in-hospital mortality rate. Patients requiring RRT had a mortality rate of 75.79%, whereas 38.76% (144/190 and 481/1241, respectively) of those who did not require RRT (*P*<.001). This finding is similar to that of a study performed by Lauridsen et al [7], which revealed that RRT in patients with CS-AMI was associated with worse mortality (62% for patients with AKI-RRT vs 36% for patients with non-AKI-RRT), along with increased long-term mortality at 5 years. This significant difference highlights the role of renal failure as a major determinant of poor outcomes in patients with CS-AMI. In addition to higher mortality, patients requiring renal support had significantly longer hospital stays (median of 10 d, IQR 4‐22 vs 6 d; *P*<.001) and higher rates of major bleeding complications (16.84% vs 7.33%; *P*<.001). Similarly, external studies have shown a longer length of stay; for example, Vallabhajosula et al [9] reported that patients with CS-AMI requiring hemodialysis had a significantly longer length of stay (mean 18, SD 19 d vs mean 9, SD 10 d). These adverse outcomes likely reflect the increased severity of illness in patients requiring RRT, as well as the risks associated with invasive procedures and anticoagulation in this population.

Other clinical outcomes, such as stroke and bleeding complications, were also more frequent in patients who required RRT. The incidence of cerebrovascular accidents was 11.05% in the renal support group, whereas it was 4.92% in those not requiring RRT (*P*=.001). Major bleeding occurred in 16.84% (32/190) of patients receiving renal support, which was more than double the rate in those who did not receive RRT (91/1241, 7.33%; *P*<.001). Freund and colleagues reported from a subanalysis of the CULPRIT-SHOCK trial that bleeding events were common in patients with infarct-related CS (21.5%), particularly in those with MCS, which is often used with renal support [24]. These complications likely contribute to the higher mortality and morbidity in this group, highlighting the complexity of managing these critically ill patients.

### LASSO Regression

LASSO regression is used in this study to handle the large number of potential predictors and avoid overfitting. By penalizing the absolute size of the regression coefficients, LASSO effectively selects the most relevant variables, making it an ideal tool for this analysis. LASSO identified several predictors of renal support in patients with CS-AMI. Key predictors included lower creatinine clearance, advanced SCAI stage, and multivessel coronary artery disease. Previous studies, such as that by Marenzi et al [8], have established that mechanical ventilation (OR 22.6, 95% CI 14.2‐36.0) and admission creatinine >1.5 mg/dL (OR 16.9, 95% CI 10.4‐27.3) were predictors of renal support. Moreover, Vallabhajosyula et al [9] reported that older age and greater comorbidity burden were associated with AKI and subsequent hemodialysis in patients with CS-AMI.

Contrary to expectations, NSTEMI presentation was associated with a higher risk of RRT compared to STEMI. This counterintuitive finding may reflect selection bias, where patients with NSTEMI developing CS represent a particularly high-risk subset with more comorbidities (diabetes and PAD) and chronic cardiac dysfunction. Alternatively, competing mortality risk may play a role, as patients with STEMI with severe shock may experience early death before developing AKI requiring RRT. This finding warrants further investigation in prospective studies and may indicate that patients with NSTEMI may benefit from more aggressive renal-protective strategies. This finding may also reflect differences in patient management strategies or in the underlying pathophysiology of AKI across subgroups. Patients with STEMI might benefit from more targeted revascularization strategies to mitigate renal injury. This is further supported by the CULPRIT-SHOCK trial, which demonstrated that, compared with immediate multivessel PCI, PCI of the culprit lesion was associated with a lower 30-day risk of death or severe renal failure requiring RRT [25].

A notable divergence exists between the variable importance rankings from the LASSO model and the significance findings from the cluster-robust logistic regression. This apparent contradiction reflects fundamental differences in the purpose and methodology of the 2 approaches rather than a true inconsistency. The LASSO model is optimized for prediction: it assigns coefficient weights based on the variable’s contribution to discriminating between patients who will and will not require RRT across the entire dataset, without regard to statistical significance. The cluster-robust logistic regression, by contrast, is designed for inference: it estimates the independent association of each variable with the outcome while explicitly accounting for the nonindependence of observations within the same center. The application of cluster-robust (sandwich) standard errors inflates the variance of coefficient estimates to correct for within-center correlation, which reduces statistical power. The 2 analyses are complementary rather than contradictory, and the clinical utility of the nomogram is not diminished by the non-significance of individual predictors in the inferential model.

### Clinical Implications

Our prediction model, presented as a user-friendly nomogram (Figure 4), is intended for use immediately post angiography, once the coronary anatomy has been defined. This represents a critical decision point in the management of CS-AMI, where clinicians must rapidly decide on the aggressiveness of hemodynamic support, the initiation of renal-protective strategies, and the need for early nephrology consultation. This early identification, aided by this tool, can trigger a cascade of preventative measures, including the avoidance of nephrotoxic agents, careful fluid management, and early involvement of a multidisciplinary team, including nephrologists, which has been shown to improve outcomes and mortality [10,11]. Finally, these data align with the growing evidence that early RRT initiation improves outcomes in selected patients.

### Limitations

Several limitations warrant consideration. First, our complete-case analysis excluded patients with missing data, which may introduce selection bias. Second, the Cockcroft-Gault equation was used to estimate creatinine clearance; this formula assumes a steady-state serum creatinine level, which is rarely the case in the acute, dynamic setting of CS and may therefore underestimate the true degree of renal impairment. Third, the inclusion of the number of diseased vessels, an angiographic variable, means the model is intended for postcatheterization risk assessment and is not applicable for precatheterization risk stratification in the emergency department. Fourth, we did not perform a competing risks analysis to account for in-hospital mortality as a competing event for RRT because our registry lacks precise timing data for both events. Fifth, although the model was validated on an internal testing set and via bootstrapping, external validation in an independent cohort is needed before widespread clinical adoption. Sixth, a further limitation concerns the treatment of SCAI shock stage in the predictive model. The LASSO model retains a single continuous coefficient for SCAI stage, and the nomogram plots the stages on a single ordinal axis. This approach assumes equidistant and linear increments in risk between consecutive stages, which may not accurately reflect the disproportionate increase in RRT risk. However, the SCAI stage was included in the model as a single variable to simplify the nomogram for clinical use and to account for the small number of events in some SCAI stages. Finally, the wide confidence intervals observed for the SCAI stage comparisons are attributable to sparse outcome data in the reference category (stage B).

### Conclusions

The study highlights significant differences in baseline characteristics and clinical outcomes between patients with CS who required renal support and those who did not. Key findings indicate that patients who needed renal support were older, had a greater prevalence of comorbidities, and presented with more severe clinical conditions, including higher rates of anterior STEMI and RV dysfunction. The analysis demonstrated that patients requiring renal replacement therapy had markedly higher hospital mortality rates and longer hospital stays, underscoring the gravity of their condition. Furthermore, the study identified critical predictors for RRT, including baseline creatinine clearance and advanced SCAI stage.

The development and internal validation of a 15-variable model allows accurate prediction of the need for RRT in patients with CS-AMI using only baseline data. Overall, these findings emphasize the importance of early identification and management of high-risk patients with CS to improve outcomes and tailor interventions more effectively. Further research may enhance the predictive capabilities and clinical applicability of these findings. Moreover, multidisciplinary management of CS, including nephrologists, is warranted.

## Supplementary material

10.2196/79678 Multimedia Appendix 1 Variables identified by Least Absolute Shrinkage and Selection Operator to be associated with renal replacement therapy.

10.2196/79678 Multimedia Appendix 2 Calibration plots before (left, blue) and after (right, green) Platt scaling. Each point represents a decile of predicted risk. Dashed line: perfect calibration. Platt scaling improved agreement between predicted and observed renal replacement therapy probabilities on the testing set (n=287).

10.2196/79678 Multimedia Appendix 3 Bootstrap validation results (1000 iterations) showing the distribution of area under the receiver operating characteristic curve values from the training set (n=1072). Mean bootstrap area under the receiver operating characteristic curve 0.745 (red dashed line); 95% CI 0.730-0.756 (green dotted lines). The narrow CI demonstrates stable model performance.

## Abbreviations

AKI: acute kidney injury
AMI: acute myocardial infarction
AUC: area under the receiver operating characteristic curve
BARC: Bleeding Academic Research Consortium
CABG: coronary artery bypass grafting
CS: cardiogenic shock
CS-AMI: cardiogenic shock secondary to acute myocardial infarction
CVA: cerebrovascular accident
ELAIN: Early versus Late Initiation of RRT in Critically Ill Patients with Acute Kidney Injury
Gulf-CS: Gulf Cardiogenic Shock
IRB: Institutional Review Board
LASSO: Least Absolute Shrinkage and Selection Operator
MCS: mechanical circulatory support
NSTEMI: non–ST-elevation myocardial infarction
OR: odds ratio
PAD: peripheral arterial disease
PCI: percutaneous coronary intervention
RRT: renal replacement therapy
RV: right ventricular
SCAI: Society for Cardiovascular Angiography and Interventions
SCAI-CSWG: Society for Cardiovascular Angiography and Interventions Cardiogenic Shock Working Group
STEMI: ST-elevation myocardial infarction
VIF: variance inflation factor
