# Supplementary material for: Postangiography Prediction of Renal Replacement Therapy in Acute Myocardial Infarction–Related Cardiogenic Shock: Least Absolute Shrinkage and Selection Operator Nomogram Development and Validation
Source: JMIR Cardio. 2026 May 20;10:e79678. doi: 10.2196/79678 (PMC13189367; doi:10.2196/79678)
Supplement: Multimedia Appendix 1 [file cardio-v10-e79678-s001.docx]

**Table S1.** Variables identified by LASSO to be associated with renal replacement therapy.

| **Variable** | **Coefficient** |
| --- | --- |
| Creatinine clearance | -0.456 |
| SCAI stage | 0.389 |
| Number of vessels | 0.259 |
| Non-ST-elevation myocardial infarction | 0.234 |
| Male | 0.228 |
| Right ventricle dysfunction | 0.159 |
| Age | 0.141 |
| Hemoglobin | -0.119 |
| Cardiac arrest | 0.097 |
| Bicarbonate | -0.079 |
| Obesity | 0.075 |
| History of percutaneous intervention | -0.069 |
| Potassium | 0.0470 |
| History of coronary artery bypass grafting | -0.021 |
| Diabetes | 0.007 |

*Abbreviations: LASSO = least absolute shrinkage and selection operator; NSTEMI = non-ST-elevation myocardial infarction; OR = odds ratio; PCI = percutaneous coronary intervention; RV = right ventricular; SCAI = Society for Cardiovascular Angiography and Interventions; SD = standard deviation; STEMI = ST-elevation myocardial infarction.*
