# Supplementary figures and images for: Postangiography Prediction of Renal Replacement Therapy in Acute Myocardial Infarction–Related Cardiogenic Shock: Least Absolute Shrinkage and Selection Operator Nomogram Development and Validation
Source: JMIR Cardio. 2026 May 20;10:e79678. doi: 10.2196/79678 (PMC13189367; doi:10.2196/79678)

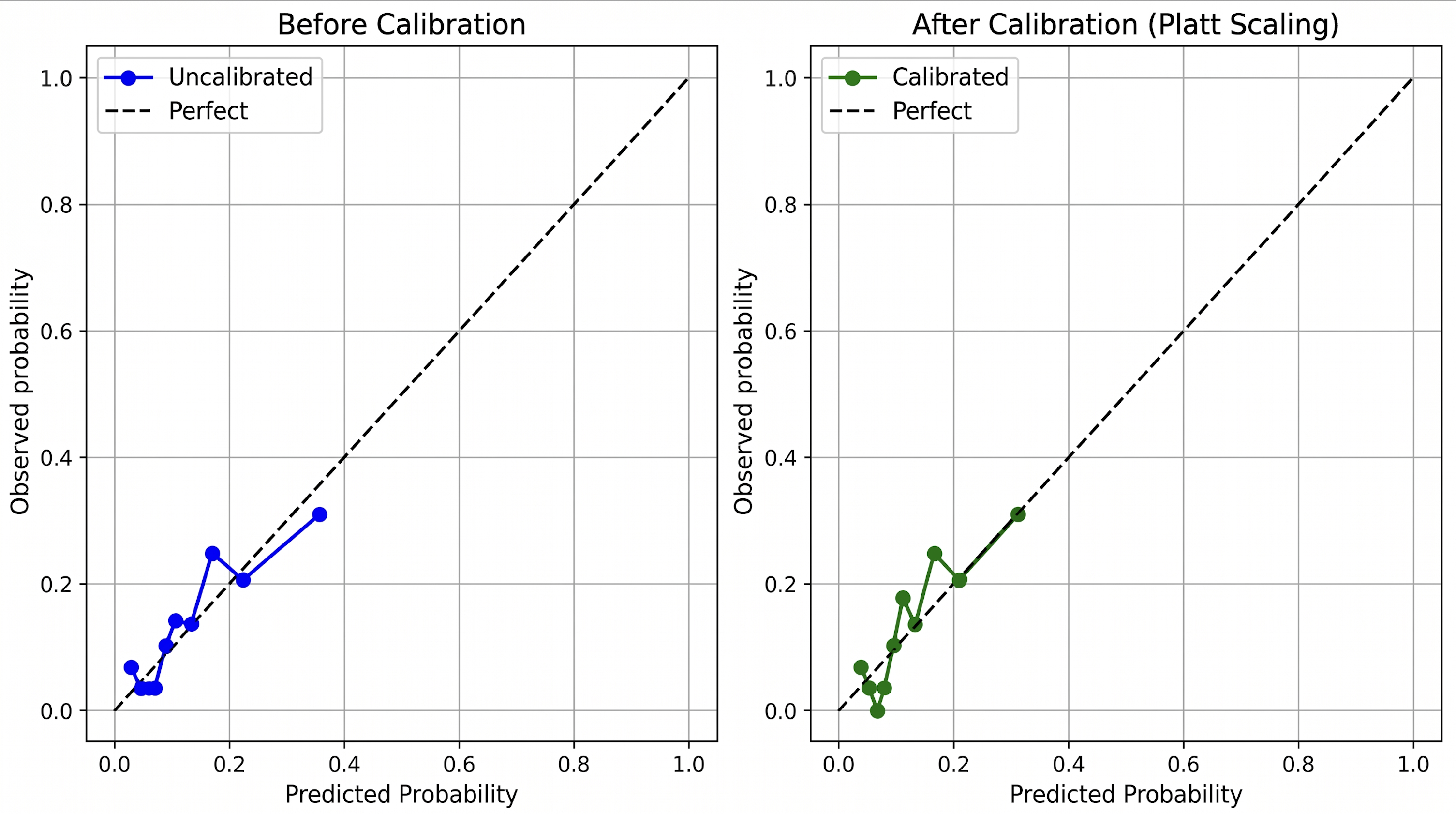

Supplement: Multimedia Appendix 2 [file cardio-v10-e79678-s002.png]

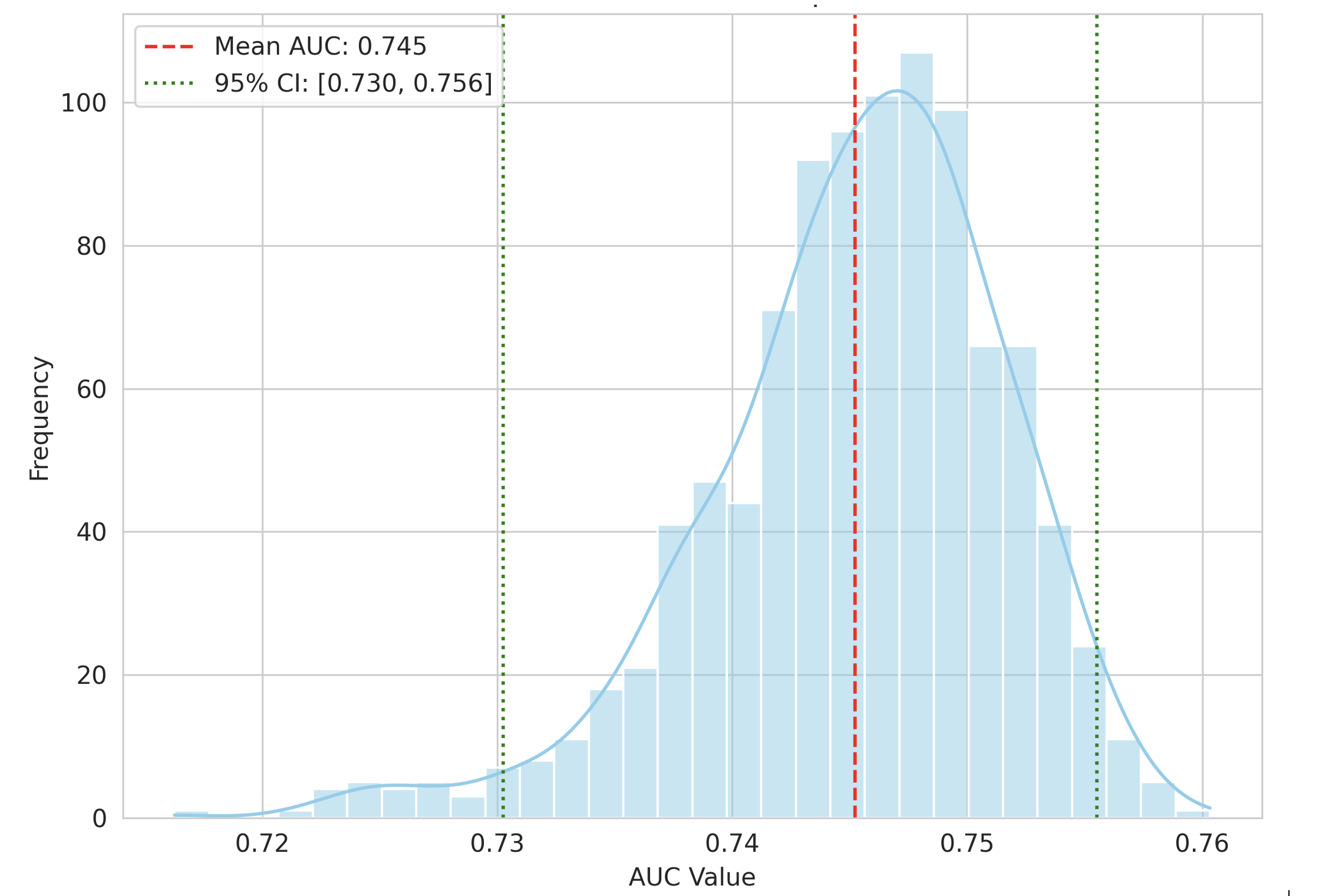

Supplement: Multimedia Appendix 3 [file cardio-v10-e79678-s003.png]
